# 10-(2-Pyrid­yloxy)phenanthren-9-ol

**DOI:** 10.1107/S1600536810033489

**Published:** 2010-08-28

**Authors:** C. A. M. A. Huq, S. Sivakumar, M. NizamMohideen

**Affiliations:** a Department of Chemistry, The New College (Autonomous), Chennai 600 014, India; b Department of Physics, The New College (Autonomous), Chennai 600 014, India

## Abstract

In the title compound, C_19_H_13_NO_2_, the pyridyl ring makes a dihedral angle of 87.04 (6)° with the plane of the phenanthrene ring system. In the crystal, mol­ecules are linked through weak inter­molecular C—H⋯O hydrogen bonds and C—H⋯π inter­actions.

## Related literature

For the biological activity of heterocyclic compounds containing a pyridine ring, see: Amr & Abdulla (2006 ▶); Borgna *et al.* (1993 ▶); Goda *et al.* (2004 ▶); Kamal *et al.* (2007 ▶). For related structures, see: Krivopalov & Shkurko (2005 ▶); Li & Flood (2008 ▶); Meudtner & Hecht (2008 ▶); Richardson *et al.* (2008 ▶); Schweinfurth *et al.* (2008 ▶).
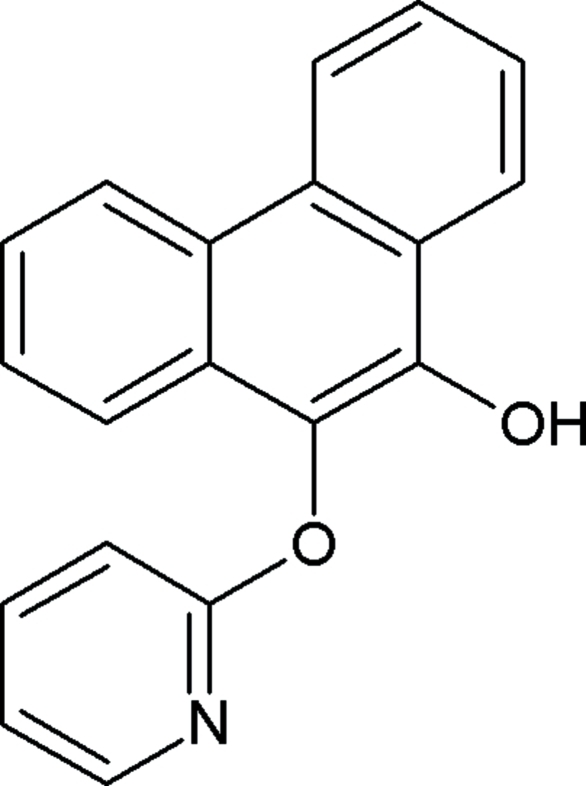


## Experimental

### 

#### Crystal data

C_19_H_13_NO_2_
*M* *_r_* = 287.30
Monoclinic, 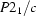

*a* = 8.9379 (6) Å
*b* = 8.6433 (10) Å
*c* = 18.389 (3) Å
β = 96.088 (8)°
*V* = 1412.6 (3) Å^3^
*Z* = 4
Cu *K*α radiation
μ = 0.71 mm^−1^
*T* = 293 K
0.3 × 0.25 × 0.2 mm

#### Data collection

Enraf–Nonius CAD-4 diffractometer
2530 measured reflections
2384 independent reflections
1828 reflections with *I* > 2σ(*I*)
*R*_int_ = 0.036
2 standard reflections every 200 reflections  intensity decay: none

#### Refinement

*R*[*F* ^2^ > 2σ(*F* ^2^)] = 0.054
*wR*(*F* ^2^) = 0.158
*S* = 1.07
2384 reflections
200 parameters
H-atom parameters constrained
Δρ_max_ = 0.34 e Å^−3^
Δρ_min_ = −0.22 e Å^−3^

### 

Data collection: *CAD-4 EXPRESS* (Enraf–Nonius, 1994 ▶); cell refinement: *CAD-4 EXPRESS*; data reduction: *XCAD4* (Harms & Wocadlo, 1995 ▶); program(s) used to solve structure: *SHELXS97* (Sheldrick, 2008 ▶); program(s) used to refine structure: *SHELXL97* (Sheldrick, 2008 ▶); molecular graphics: *ORTEP-3* (Farrugia, 1997 ▶) and *PLATON* (Spek, 2009 ▶); software used to prepare material for publication: *SHELXL97* and *PLATON*.

## Supplementary Material

Crystal structure: contains datablocks global, I. DOI: 10.1107/S1600536810033489/lx2166sup1.cif

Structure factors: contains datablocks I. DOI: 10.1107/S1600536810033489/lx2166Isup2.hkl

Additional supplementary materials:  crystallographic information; 3D view; checkCIF report

## Figures and Tables

**Table 1:** Hydrogen-bond geometry (Å, °) *Cg*1 and *Cg*2 are the centroids of the N1/C1–C5 and C6/C7/C12/C13/C18/C19 rings, respectively.

*D*—H⋯*A*	*D*—H	H⋯*A*	*D*⋯*A*	*D*—H⋯*A*
C3—H3⋯O2^i^	0.93	2.59	3.475 (3)	159
C10—H10⋯*Cg*1^ii^	0.93	2.86	3.691 (3)	150
C15—H15⋯*Cg*2^iii^	0.93	2.80	3.537 (3)	137

Symmetry codes: (i) 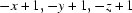; (ii) 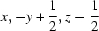; (iii) 
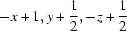
.

**Fig. 1.**
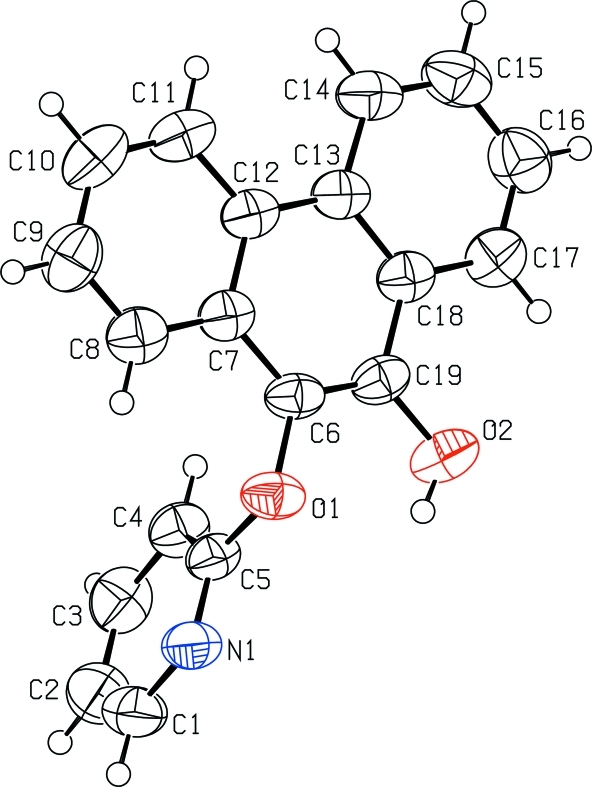
The molecular structure of the title compound with the atom numbering scheme. Displacement ellipsoids are drawn at the 50% probability level. H atoms are presented as a small spheres of arbitrary radius.

**Fig. 2.**
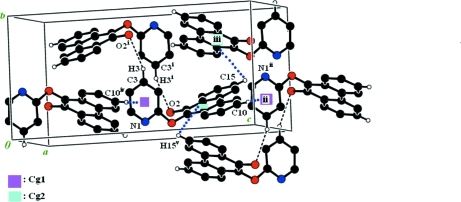
C—H···O and C—H···π interactions (dotted lines) in the crystal structure of the title compound. Cg denotes the ring centroids. [Symmetry codes: (i) -x + 1, -y + 1, - z + 1; (ii) x, -y + 1/2, z + 1/2; (iii) - x + 2, y + 1/2, - z + 3/2; (iv) x, - y + 1/2, z - 1/2; (v) - x + 2, y - 1/2, - z + 3/2.]

## supplementary crystallographic information

### Comment

Heterocyclic compounds containing the pyridine ring are reported to possess a diverse range of biological activities such as antimicrobial, antitumor and anti–inflammatory (Amr & Abdulla, 2006; Borgna *et al.*, 1993; Goda *et al.*, 2004; Kamal *et al.*, 2007) properties. Pyridyl functionalized 1,2,3–triazoles have begun attracting significant attention in a range of areas including anion recognition (Li & Flood, 2008), stimuli responsive foldamers (Meudtner & Hecht, 2008), drug discovery (Krivopalov & Shkurko, 2005) and coordination chemistry (Richardson *et al.*, 2008; Schweinfurth *et al.*, 2008). Against this background the structure of the title compound was determined by X–ray diffraction. Here we report the crystal structure of the title compound (Fig. 1).

The phenanthrene plane is essentially planar, with a mean deviation of 0.011 (2) |%A from the least-squares plane defined by the  fourteen constituent atoms. The dihedral angle formed by the phenanthrene plane and the pyridyl ring is  87.04 (6)°. The crystal packing (Fig. 2) is stabilized by a weak intermolecular C—H···O hydrogen bond between the pyidyl H atom and the oxygen of the hydroxyl group, with a C3—H3···O2^i^ (Table 1). The crystal packing (Fig. 2) is further stabilized by two intermolecular C—H···π interactions; the first one between the benzene H atom of the phenanthrene unit and the pyridyl ring, with a C10—H10···*Cg*1, the second one between the benzene H atom of the phenanthrene unit and the central benzene ring of a neighbouring molecule, with a C15—H15···*Cg*2 (Table 1; *Cg*1 and *Cg*2 are the centroids of the N1/C1—C5 pyridyl ring and the C6/C7/C12/C13/C18/C19 benzene ring, respectively).

### Experimental

To a solution of 2–pyridyl magnesium bromide in dry THF at 273° under nitrogen atomsphere, solution of phenanthren-9,10-dione in dry THF was added dropwise. After the addition, the mixture was stirred at room temp under nitrogen atmosphere for 3 h. After the completion of reaction as evidenced by TLC, the reaction was quenched with saturated solution of ammonium chloride and the mixture was extracted into diethyl ether. The organic layer was concentrated at reduced pressure. The residue was purified by coloumn chromotography (hexane-ethyl acetate, 9:1 *v*/*v*) to afford the title compound as a pale yellow solid (yield 75%, m.p. 418° K). Single crystals suitable for X–ray diffraction was recrystallized from mixture of dichlromehane-hexane (8: 2 *v*/*v*) as solvent.

### Refinement

All H atoms were positioned geometrically, with C—H = 0.93–0.98 Å and constrained to ride on their parent atoms, with *U*_iso_(H) = *xU*_eq_(C, N), where *x* = 1.5 for methyl H and *x* = 1.2 for all H atoms.

### Crystal data

**Table:**

C_19_H_13_NO_2_	*F*(000) = 600
*M**_r_* = 287.30	*D*_x_ = 1.351 Mg m^−^^3^
Monoclinic, *P*2_1_/*c*	Cu *K*α radiation, λ = 1.54180 Å
Hall symbol: -P 2ybc	Cell parameters from 25 reflections
*a* = 8.9379 (6) Å	θ = 20–32°
*b* = 8.6433 (10) Å	µ = 0.71 mm^−^^1^
*c* = 18.389 (3) Å	*T* = 293 K
β = 96.088 (8)°	Block, yellow
*V* = 1412.6 (3) Å^3^	0.3 × 0.25 × 0.2 mm
*Z* = 4	

### Data collection

**Table:**

Enraf–Nonius CAD-4 diffractometer	*R*_int_ = 0.036
Radiation source: fine-focus sealed tube	θ_max_ = 64.9°, θ_min_ = 4.8°
graphite	*h* = 0→10
ω–2θ scan	*k* = 0→10
2530 measured reflections	*l* = −21→21
2384 independent reflections	2 standard reflections every 100 reflections
1828 reflections with *I* > 2σ(*I*)	intensity decay: none

### Refinement

**Table:**

Refinement on *F*^2^	Secondary atom site location: difference Fourier map
Least-squares matrix: full	Hydrogen site location: difference Fourier map
*R*[*F*^2^ > 2σ(*F*^2^)] = 0.054	H-atom parameters constrained
*wR*(*F*^2^) = 0.158	*w* = 1/[σ^2^(*F*_o_^2^) + (0.0879*P*)^2^ + 0.3509*P*] where *P* = (*F*_o_^2^ + 2*F*_c_^2^)/3
*S* = 1.07	(Δ/σ)_max_ < 0.001
2384 reflections	Δρ_max_ = 0.34 e Å^−^^3^
200 parameters	Δρ_min_ = −0.22 e Å^−^^3^
0 restraints	Extinction correction: *SHELXL97* (Sheldrick, 2008), Fc^*^=kFc[1+0.001xFc^2^λ^3^/sin(2θ)]^-1/4^
Primary atom site location: structure-invariant direct methods	Extinction coefficient: 0.0161 (14)

### Special details

**Table:**

Geometry. All e.s.d.'s (except the e.s.d. in the dihedral angle between two l.s. planes) are estimated using the full covariance matrix. The cell e.s.d.'s are taken into account individually in the estimation of e.s.d.'s in distances, angles and torsion angles; correlations between e.s.d.'s in cell parameters are only used when they are defined by crystal symmetry. An approximate (isotropic) treatment of cell e.s.d.'s is used for estimating e.s.d.'s involving l.s. planes.
Refinement. Refinement of *F*^2^ against ALL reflections. The weighted *R*-factor *wR* and goodness of fit *S* are based on *F*^2^, conventional *R*-factors *R* are based on *F*, with *F* set to zero for negative *F*^2^. The threshold expression of *F*^2^ > σ(*F*^2^) is used only for calculating *R*-factors(gt) *etc*. and is not relevant to the choice of reflections for refinement. *R*-factors based on *F*^2^ are statistically about twice as large as those based on *F*, and *R*- factors based on ALL data will be even larger.

### Fractional atomic coordinates and isotropic or equivalent isotropic displacement parameters (Å^2^)

**Table:**

	*x*	*y*	*z*	*U*_iso_*/*U*_eq_	
N1	0.28079 (19)	0.0991 (2)	0.50744 (9)	0.0552 (5)	
O1	0.48937 (16)	0.09045 (15)	0.58522 (7)	0.0539 (4)	
O2	0.75971 (19)	0.19460 (18)	0.54090 (8)	0.0639 (5)	
H2	0.6870	0.1572	0.5161	0.096*	
C1	0.1729 (3)	0.1774 (3)	0.46753 (13)	0.0687 (7)	
H1	0.0995	0.1213	0.4392	0.082*	
C2	0.1638 (3)	0.3354 (3)	0.46580 (14)	0.0734 (7)	
H2A	0.0876	0.3854	0.4364	0.088*	
C3	0.2706 (3)	0.4179 (3)	0.50877 (13)	0.0675 (7)	
H3	0.2666	0.5254	0.5094	0.081*	
C4	0.3832 (3)	0.3413 (3)	0.55083 (12)	0.0566 (6)	
H4	0.4566	0.3949	0.5803	0.068*	
C5	0.3832 (2)	0.1817 (2)	0.54766 (10)	0.0470 (5)	
C6	0.5977 (2)	0.1611 (2)	0.63482 (10)	0.0474 (5)	
C7	0.5655 (2)	0.1757 (2)	0.70924 (11)	0.0480 (5)	
C8	0.4266 (3)	0.1302 (3)	0.73138 (13)	0.0596 (6)	
H8	0.3536	0.0884	0.6972	0.072*	
C9	0.3968 (3)	0.1465 (3)	0.80255 (14)	0.0690 (7)	
H9	0.3042	0.1163	0.8166	0.083*	
C10	0.5066 (3)	0.2086 (3)	0.85376 (14)	0.0711 (7)	
H10	0.4870	0.2197	0.9021	0.085*	
C11	0.6431 (3)	0.2533 (3)	0.83355 (12)	0.0619 (6)	
H11	0.7152	0.2934	0.8687	0.074*	
C12	0.6769 (2)	0.2399 (2)	0.76057 (11)	0.0493 (5)	
C13	0.8179 (2)	0.2907 (2)	0.73679 (11)	0.0501 (5)	
C14	0.9345 (3)	0.3569 (3)	0.78452 (13)	0.0637 (6)	
H14	0.9197	0.3717	0.8333	0.076*	
C15	1.0677 (3)	0.3998 (3)	0.76122 (15)	0.0724 (7)	
H15	1.1428	0.4421	0.7942	0.087*	
C16	1.0924 (3)	0.3807 (3)	0.68872 (15)	0.0711 (7)	
H16	1.1837	0.4107	0.6731	0.085*	
C17	0.9837 (3)	0.3184 (3)	0.64021 (13)	0.0610 (6)	
H17	1.0013	0.3056	0.5916	0.073*	
C18	0.8444 (2)	0.2729 (2)	0.66282 (11)	0.0499 (5)	
C19	0.7291 (2)	0.2060 (2)	0.61153 (10)	0.0490 (5)	

### Atomic displacement parameters (Å^2^)

**Table:**

	*U*^11^	*U*^22^	*U*^33^	*U*^12^	*U*^13^	*U*^23^
N1	0.0622 (11)	0.0553 (11)	0.0456 (10)	0.0030 (9)	−0.0056 (8)	−0.0065 (8)
O1	0.0661 (9)	0.0412 (8)	0.0506 (8)	0.0005 (6)	−0.0119 (7)	−0.0040 (6)
O2	0.0903 (11)	0.0595 (9)	0.0425 (8)	−0.0129 (8)	0.0099 (7)	−0.0048 (7)
C1	0.0683 (14)	0.0799 (17)	0.0541 (13)	0.0067 (13)	−0.0111 (11)	−0.0061 (12)
C2	0.0766 (16)	0.0800 (18)	0.0613 (15)	0.0221 (14)	−0.0028 (12)	0.0118 (13)
C3	0.0822 (16)	0.0522 (14)	0.0689 (15)	0.0120 (12)	0.0120 (13)	0.0076 (11)
C4	0.0687 (14)	0.0476 (12)	0.0531 (12)	0.0002 (10)	0.0046 (10)	−0.0013 (10)
C5	0.0572 (12)	0.0475 (11)	0.0356 (10)	0.0030 (9)	0.0022 (8)	−0.0007 (8)
C6	0.0610 (12)	0.0360 (10)	0.0427 (10)	−0.0001 (9)	−0.0062 (9)	−0.0026 (8)
C7	0.0594 (12)	0.0371 (10)	0.0463 (11)	0.0049 (9)	0.0004 (9)	0.0001 (8)
C8	0.0681 (14)	0.0507 (12)	0.0596 (13)	−0.0014 (11)	0.0040 (11)	−0.0012 (10)
C9	0.0797 (16)	0.0602 (15)	0.0705 (16)	−0.0034 (12)	0.0234 (13)	0.0006 (12)
C10	0.1028 (19)	0.0614 (15)	0.0528 (13)	−0.0004 (14)	0.0252 (13)	−0.0022 (11)
C11	0.0860 (17)	0.0543 (13)	0.0444 (12)	−0.0012 (12)	0.0023 (11)	−0.0055 (10)
C12	0.0646 (13)	0.0387 (10)	0.0431 (11)	0.0052 (9)	−0.0009 (9)	−0.0002 (8)
C13	0.0608 (13)	0.0403 (10)	0.0471 (11)	0.0044 (9)	−0.0051 (9)	−0.0016 (9)
C14	0.0711 (15)	0.0630 (14)	0.0534 (13)	−0.0028 (12)	−0.0099 (11)	−0.0073 (11)
C15	0.0687 (15)	0.0661 (16)	0.0781 (17)	−0.0094 (13)	−0.0118 (13)	−0.0095 (13)
C16	0.0618 (14)	0.0638 (15)	0.0872 (18)	−0.0101 (12)	0.0058 (12)	−0.0036 (14)
C17	0.0701 (14)	0.0528 (13)	0.0607 (13)	−0.0049 (11)	0.0100 (11)	−0.0015 (11)
C18	0.0617 (13)	0.0386 (10)	0.0483 (11)	0.0018 (9)	0.0008 (9)	−0.0011 (8)
C19	0.0679 (13)	0.0386 (11)	0.0401 (11)	0.0022 (9)	0.0028 (9)	−0.0016 (8)

### Geometric parameters (Å, °)

**Table:**

N1—C5	1.323 (3)	C8—H8	0.9300
N1—C1	1.333 (3)	C9—C10	1.394 (4)
O1—C5	1.364 (2)	C9—H9	0.9300
O1—C6	1.398 (2)	C10—C11	1.368 (4)
O2—C19	1.359 (2)	C10—H10	0.9300
O2—H2	0.8200	C11—C12	1.411 (3)
C1—C2	1.368 (4)	C11—H11	0.9300
C1—H1	0.9300	C12—C13	1.445 (3)
C2—C3	1.372 (4)	C13—C14	1.411 (3)
C2—H2A	0.9300	C13—C18	1.413 (3)
C3—C4	1.373 (3)	C14—C15	1.359 (3)
C3—H3	0.9300	C14—H14	0.9300
C4—C5	1.381 (3)	C15—C16	1.384 (4)
C4—H4	0.9300	C15—H15	0.9300
C6—C19	1.350 (3)	C16—C17	1.359 (3)
C6—C7	1.434 (3)	C16—H16	0.9300
C7—C8	1.403 (3)	C17—C18	1.410 (3)
C7—C12	1.411 (3)	C17—H17	0.9300
C8—C9	1.370 (3)	C18—C19	1.441 (3)
			
C5—N1—C1	116.8 (2)	C11—C10—C9	120.6 (2)
C5—O1—C6	118.32 (15)	C11—C10—H10	119.7
C19—O2—H2	109.5	C9—C10—H10	119.7
N1—C1—C2	123.9 (2)	C10—C11—C12	121.5 (2)
N1—C1—H1	118.1	C10—C11—H11	119.3
C2—C1—H1	118.1	C12—C11—H11	119.3
C1—C2—C3	118.0 (2)	C7—C12—C11	117.5 (2)
C1—C2—H2A	121.0	C7—C12—C13	119.54 (19)
C3—C2—H2A	121.0	C11—C12—C13	122.99 (19)
C2—C3—C4	119.8 (2)	C14—C13—C18	117.1 (2)
C2—C3—H3	120.1	C14—C13—C12	123.0 (2)
C4—C3—H3	120.1	C18—C13—C12	119.90 (19)
C3—C4—C5	117.5 (2)	C15—C14—C13	121.9 (2)
C3—C4—H4	121.2	C15—C14—H14	119.0
C5—C4—H4	121.2	C13—C14—H14	119.0
N1—C5—O1	111.97 (18)	C14—C15—C16	120.4 (2)
N1—C5—C4	123.98 (19)	C14—C15—H15	119.8
O1—C5—C4	124.04 (18)	C16—C15—H15	119.8
C19—C6—O1	118.98 (18)	C17—C16—C15	120.2 (2)
C19—C6—C7	123.17 (19)	C17—C16—H16	119.9
O1—C6—C7	117.78 (18)	C15—C16—H16	119.9
C8—C7—C12	119.97 (19)	C16—C17—C18	120.6 (2)
C8—C7—C6	121.70 (19)	C16—C17—H17	119.7
C12—C7—C6	118.32 (19)	C18—C17—H17	119.7
C9—C8—C7	121.1 (2)	C17—C18—C13	119.8 (2)
C9—C8—H8	119.5	C17—C18—C19	120.7 (2)
C7—C8—H8	119.5	C13—C18—C19	119.50 (19)
C8—C9—C10	119.4 (2)	C6—C19—O2	123.53 (19)
C8—C9—H9	120.3	C6—C19—C18	119.55 (18)
C10—C9—H9	120.3	O2—C19—C18	116.91 (18)
			
C5—N1—C1—C2	0.4 (3)	C10—C11—C12—C7	−1.2 (3)
N1—C1—C2—C3	−1.2 (4)	C10—C11—C12—C13	177.9 (2)
C1—C2—C3—C4	1.0 (4)	C7—C12—C13—C14	179.54 (19)
C2—C3—C4—C5	0.0 (3)	C11—C12—C13—C14	0.5 (3)
C1—N1—C5—O1	−178.97 (18)	C7—C12—C13—C18	−1.3 (3)
C1—N1—C5—C4	0.6 (3)	C11—C12—C13—C18	179.68 (19)
C6—O1—C5—N1	−174.68 (17)	C18—C13—C14—C15	−1.1 (3)
C6—O1—C5—C4	5.7 (3)	C12—C13—C14—C15	178.1 (2)
C3—C4—C5—N1	−0.9 (3)	C13—C14—C15—C16	0.7 (4)
C3—C4—C5—O1	178.70 (18)	C14—C15—C16—C17	−0.3 (4)
C5—O1—C6—C19	−91.9 (2)	C15—C16—C17—C18	0.3 (4)
C5—O1—C6—C7	90.9 (2)	C16—C17—C18—C13	−0.7 (3)
C19—C6—C7—C8	178.89 (19)	C16—C17—C18—C19	−179.7 (2)
O1—C6—C7—C8	−4.0 (3)	C14—C13—C18—C17	1.1 (3)
C19—C6—C7—C12	−0.2 (3)	C12—C13—C18—C17	−178.16 (19)
O1—C6—C7—C12	176.95 (16)	C14—C13—C18—C19	−179.92 (19)
C12—C7—C8—C9	−0.2 (3)	C12—C13—C18—C19	0.8 (3)
C6—C7—C8—C9	−179.3 (2)	O1—C6—C19—O2	3.9 (3)
C7—C8—C9—C10	−0.3 (4)	C7—C6—C19—O2	−179.02 (18)
C8—C9—C10—C11	0.0 (4)	O1—C6—C19—C18	−177.34 (17)
C9—C10—C11—C12	0.7 (4)	C7—C6—C19—C18	−0.2 (3)
C8—C7—C12—C11	0.9 (3)	C17—C18—C19—C6	178.89 (19)
C6—C7—C12—C11	−179.97 (18)	C13—C18—C19—C6	−0.1 (3)
C8—C7—C12—C13	−178.16 (18)	C17—C18—C19—O2	−2.2 (3)
C6—C7—C12—C13	0.9 (3)	C13—C18—C19—O2	178.76 (17)

### Hydrogen-bond geometry (Å, °)

**Table:**

Cg1 and Cg2 are the centroids of the N1/C1–C5 and C6/C7/C12/C13/C18/C19 rings, respectively.

**Table:**

*D*—H···*A*	*D*—H	H···*A*	*D*···*A*	*D*—H···*A*
C3—H3···O2^i^	0.93	2.59	3.475 (3)	159.
C10—H10···Cg1^ii^	0.93	2.86	3.691 (3)	150
C15—H15···Cg2^iii^	0.93	2.80	3.537 (3)	137

Symmetry codes: (i) −*x*+1, −*y*+1, −*z*+1; (ii) *x*, −*y*+1/2, *z*−1/2; (iii) −*x*+1, *y*+1/2, −*z*+1/2.
